# Epidemiology of Foodborne Botulism Outbreaks in Romania, 2007–2024

**DOI:** 10.3390/microorganisms14040819

**Published:** 2026-04-02

**Authors:** Bianca Georgiana Enciu, Rodica Popescu, Alina Daniela Zaharia, Barbara Schimmer, Daniela Pițigoi, Anca Mirela Sîrbu, Adriana Pistol

**Affiliations:** 1 Department of Epidemiology II, Carol Davila University of Medicine and Pharmacy, 050463 Bucharest, Romania; bianca.enciu@umfcd.ro (B.G.E.); anca.sirbu@insp.gov.ro (A.M.S.); adriana.pistol@umfcd.ro (A.P.); 2 National Centre for Communicable Diseases Surveillance and Control (CNSCBT), National Institute of Public Health, 050463 Bucharest, Romania; rodica.popescu@insp.gov.ro; 3 ECDC Fellowship Programme, Field Epidemiology Path (EPIET), European Centre for Disease Prevention and Control (ECDC), 16973 Stockholm, Sweden; 4 Centre for Infectious Diseases Control, National Institute for Public Health and the Environment, 3720 BA Bilthoven, The Netherlands; barbara.schimmer@rivm.nl; 5 Department of Epidemiology I, Carol Davila University of Medicine and Pharmacy, 021105 Bucharest, Romania; daniela.pitigoi@umfcd.ro; 6 National Institute for Infectious Disease “Prof. Dr. Matei Balș”, 021105 Bucharest, Romania

**Keywords:** outbreak, surveillance, botulinum neurotoxin, preparedness, Romania

## Abstract

Foodborne botulism, caused by ingestion of pre-formed botulinum neurotoxin, is the most common form of botulism. While large outbreaks linked to commercial foods are rare, smaller outbreaks associated with home-processed products are more frequent, reflecting local dietary habits and traditional preservation practices. The aim of this paper is to provide a public health overview of reported foodborne botulism outbreaks in Romania over an 18-year period to raise awareness among clinicians and public health officials. Between 2007 and 2024, a total of 337 foodborne botulism cases were reported in Romania, of which 43% (147) were related to 55 outbreaks (median number of cases per outbreak: 2; IQR: 2–3). Most outbreaks were reported in Bihor County (11 outbreaks with 29 cases) and its neighboring county, Satu Mare (seven outbreaks, accounting for a total number of 20 cases). Outbreak-related cases were observed in younger persons with a median age of 31 years (compared to 45 years for sporadic cases) and were statistically significantly associated with consumption of pork products (*p* < 0.001). Fifteen deaths occurred (case fatality ratio: 4%), including three outbreak-related cases (case fatality ratio: 2%). These findings highlight the ongoing public health challenge of foodborne botulism in Romania and the need for robust surveillance, targeted educational initiatives in high-incidence counties to deliver information about safe food preparation and preservation practices, and the continuous availability of botulinum antitoxin supplies.

## 1. Introduction

Foodborne botulism occurs worldwide, and it is one of the most prevalent forms of botulism, caused by the ingestion of pre-formed botulinum neurotoxin (BoNT) produced mainly by *Clostridium botulinum* [1]. These bacteria develop and produce toxins in food items that have low oxygen levels, a PH ≥ 4.5, low sugar and salt content, and are stored at temperatures more than 3 °C. The conditions are often met in home-canned, preserved or fermented food items (e.g., vegetables, meat or fish) [2,3,4]. Nine types of BoNT have been recognized (A–G, H or H/A or F/A and X), but types A, B and E are primarily associated with foodborne botulism [5,6]. Clinical manifestations of botulism usually develop 12–36 h after exposure, although the incubation period may range from 4 h to 8 days [2,7]. Early symptoms include gastrointestinal discomfort and xerostomia, followed by typical signs of botulism such as diplopia, blurred vision, dysphagia, bulbar weakness, and eventually symmetrical peripheral paralysis affecting both voluntary and autonomic muscles [7]. Although the progression of paralysis in botulism patients has been described as unique and very easily recognizable, in practice, botulism is often initially misdiagnosed, especially among children and adolescents [8]. Early clinical recognition based on patient anamnesis and thorough neurological examination is essential for case management and for improving survival rates, considering the current case fatality rates that are between 3% and 10% [2,5,8,9,10,11,12].

Delays in botulism diagnosis, laboratory confirmation and testing limitations might trigger delays in public health interventions, allowing contaminated food to be further consumed and increasing the risk of outbreaks [13]. Large foodborne botulism outbreaks are exceedingly rare and are typically linked to commercially prepared foods, while smaller outbreaks are more common and are most often linked to the consumption of home-processed food [14,15].

In Romania, high incidence of foodborne botulism cases, either sporadically or as part of outbreaks, is related to the consumption of home-made pork products, typically resulting from the customary slaughter of a backyard pig during winter as Christmas celebration activity or with the slaughter of a second pig in June or July, when the winter supply of pork has been depleted [6,16].

The aim of this paper is to provide a public health overview of reported foodborne botulism outbreaks in Romania over an 18-year period to raise awareness among clinicians and public health officials.

## 2. Materials and Methods

Botulism surveillance in Romania: A passive, case-based surveillance system for botulism, in line with the European Union (EU) standards, established by law, has been operational in Romania since 2007 [17,18,19,20]. According to these regulatory acts, all healthcare providers are responsible for notifying the county public health authority of suspected botulism cases. They should provide this notification over the phone immediately after detection. The public health authorities’ staff are also required to notify the National Institute of Public Health and to conduct an epidemiological investigation for each case [19,20]. In August 2024, a specific surveillance system for botulism was implemented, aiming to standardize and strengthen data collection and the response to botulism cases; it requires the completion of a specific sheet for botulism cases in addition to the unique reporting sheet for notifiable diseases which was first approved in 2007 and further updated in 2022 [19,21].

Case and outbreak definitions: The case definition used for surveillance of foodborne botulism includes clinical criteria (signs of bilateral cranial nerve palsy, such as diplopia, blurred vision, dysphagia, or symmetrical peripheral paralysis); epidemiological criteria (exposure to contaminated food or drinking water); and laboratory criteria (detection of BoNT or BoNT-encoding genes in a clinical sample or isolation of neurotoxin-producing *clostridia*). A probable case is defined as a case that meets at least one clinical criterion and the epidemiological criterion, while a confirmed case is defined as a case meeting at least one clinical criterion and one laboratory criterion. A foodborne botulism outbreak was defined as the occurrence of at least two confirmed or probable cases linked to common exposure. If this threshold was not reached, any confirmed or probable case was classified as sporadic. Laboratory investigations: Laboratory confirmation of probable cases was carried out by the national reference laboratory for anaerobic bacteria and zoonoses. Food investigations were performed either by the national reference laboratory or by the Food Safety Authorities (FSAs), depending on the local testing capacity [21].

Data sources and analysis: We conducted a retrospective study using national botulism surveillance data collected from 2007 to 2024. Over time, botulism surveillance data were compiled into annual Excel files; these files contained demographic, clinical, laboratory and epidemiological data derived from the unique reporting sheet for notifiable disease, laboratory bulletins and investigation reports submitted by county public health authorities. As no standardized botulism investigation report existed before August 2024, reporting formats varied over time. For this study, we merged the surveillance datasets for the 18-year period and then restricted it considering the following inclusion or exclusion criteria.

Inclusion criteria: a probable or confirmed foodborne botulism case according to the case definition, for which the existing investigation reports pointed out an incriminated food vehicle, either suspected or laboratory confirmed.

Exclusion criteria: cases for which the existing investigation reports did not provide any information on incriminated food items; cases that reported symptoms and food exposures but had a negative laboratory result for a patient-collected sample.

We conducted a descriptive analysis of the notified foodborne botulism outbreaks from 2007 to 2024 and separately summarized the large foodborne botulism outbreaks, defined as outbreaks accounting for at least twice as many cases as the mean number of cases per outbreak recorded during the study period. In the end, we conducted a comparative case–case analysis between sporadic cases and outbreak-related cases.

For continuous variables we reported the mean, median and the interquartile range (IQR), while for categorical variables we reported frequencies and percentages. The Mann–Whitney U test, Pearson’s Chi-squared test, and Fisher’s exact test were applied to evaluate differences between groups, as appropriate for the type of data analyzed. Two-tailed *p* values less than 0.05 were considered statistically significant. Statistical analyses were conducted in Microsoft Excel for 365 and RStudio version 2024.12.1.

## 3. Results

### 3.1. Foodborne Botulism Outbreaks in Romania, 2007–2024

Between 2007 and 2024, a total of 337 foodborne botulism cases were reported in Romania, of which 43% (147) were related to 55 outbreaks (median number of cases per outbreak: 2; mean: 3; IQR: 2–3).

The median number of outbreaks per year was three (mean: 3; IQR: 2–5), with the highest number of outbreaks reported in 2007 and 2009 (six outbreaks/per year, accounting for 13 and 22 cases, respectively). No foodborne botulism outbreaks were notified in 2020 and 2021. The months recording the highest number of outbreaks were January (six outbreaks with 20 cases), February (six outbreaks with 18 cases), May (seven outbreaks with 24 cases), June (seven outbreaks with 18 cases), and August (seven outbreaks with 11 cases). The highest number of outbreaks was recorded in Bihor (11 outbreaks with 29 cases) and its neighboring county, Satu Mare (seven outbreaks with 20 cases, one living in Bihor). The median age of outbreak cases was 31 years (mean: 35; IQR: 23–48), with the male/female ratio being 1.45:1.

Forty-six outbreaks (86%) accounting for a total number of 127 cases (86%) were caused by pork products. Food investigation results were available for four outbreaks, two notified in Satu Mare (in 2009-BoNT type B and 2014-BoNT), one in Timiș (in 2010-BoNT type B) and one in Iași (in 2019-BoNT). The main characteristics of the 55 outbreaks recorded are listed in the Supplementary Material Table S1. Three outbreaks met the definition for large outbreaks; their characteristics are listed below.

Outbreak 1

The first large outbreak was reported in February 2009 in Argeș County. It was a point-source outbreak involving six adult cases (median age: 40 years; range: 27–52 years), all male and all hospitalized, who were confirmed to have type B BoNT. The median time from disease onset to reporting was 7 days. One death was recorded, resulting in a case fatality ratio of 17%. The outbreak involved members of the same construction team working in a rural area of Argeș County; they used to share lunches. The preliminary investigation pointed out a canned fish in oil purchased from a local supermarket as the outbreak vehicle. It was consumed by all cases three days before symptoms’ onset. No remnants of the product could be recovered due to the reporting delay. Following notification of the food safety authorities, approximately 4% of the implicated batch of canned fish in oil was tested, with all samples yielding negative results for BoNT. Considering these findings and the inconsistencies in the initial responses on food consumption, the cases were re-interviewed, and consumption of home-made pork ham was also reported. This food item was considered as the most likely outbreak vehicle. However, it was no longer available for testing.

Outbreak 2

The second large outbreak was reported in May 2009 in Satu Mare County. This was a point-source outbreak involving six cases (median age: 31 years; range: 7–59 years; male/female ratio: 1/1), who were classified as probable. No deaths were recorded. Food investigation revealed the presence of the type B BoNT in home-made pork ham.

Outbreak 3

The third large outbreak was reported in January 2024 in Timiș County. It was a point-source outbreak involving eight pediatric cases (median age: 6 years; range: 3–8 years; male/female ratio: 6/2, all hospitalized) in a household setting of a 13-member foster family. Cases initially presented with gastrointestinal symptoms that were not recognized as botulism. About two weeks later, when typical signs of botulism appeared, a diagnosis of foodborne botulism was established; laboratory testing confirmed BoNT in all cases: six with unspecified BoNT and two specifically with type B BoNT. The outbreak investigation identified home-made pork sausages received during door-to-door caroling as the likely vehicle, but they were unavailable for testing. Additionally, BoNT was not detected in any of the tested food items: available home-made pork sausages (other than those incriminated), pork cracklings, frozen pork, and chicken.

### 3.2. Comparative Analysis Between Outbreak-Related Cases and Sporadic Cases, 2007–2024

The comparative analysis between sporadic (190; 57%) and outbreak-related cases (147; 43%) showed statistically significant differences in terms of age and incriminated food items, with outbreak-related cases recorded among younger ages (median: 31; mean: 35; IQR: 23–48 vs. median: 45; mean: 44; IQR: 33–55; *p* < 0.001) and among people who consumed pork products (127; 86% vs. 122; 64%; *p* < 0.001).

Twelve out of fifteen deaths (80%) occurred among sporadic cases, resulting in a case fatality ratio of 6% for sporadic cases and 2% for outbreak-related cases (Table 1). While more sporadic cases were laboratory-investigated (175; 92% vs. 128; 87%), type B BoNT was found in most of the cases; either sporadic (151; 87%) or outbreak-related (96; 75%); type E BoNT was found in only one sporadic case.

**Figure 1:**
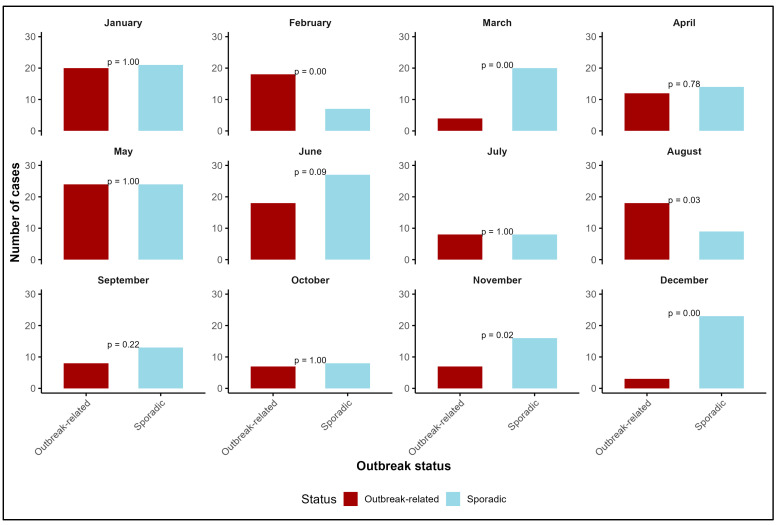
Comparative analysis of foodborne botulism cases by status and reporting month, Romania, 2007–2024 (N = 337).

Comparative distribution of cases by month showed February (18; 72% vs. 7; 28%, *p* = 0.004) and August (18; 67% vs. 9; 33%, *p* = 0.029) had a statistically significantly higher number of outbreak-related cases than sporadic cases, while in March (4; 17% vs. 2; 83%, *p* < 0.001), November (7; 30% vs. 16; 70%, *p* = 0.2) and December (3; 12% vs. 23; 88%, *p* < 0.001), there were more sporadic cases than outbreak-related cases (Figure 1).

## 4. Discussion

Foodborne botulism, although rare in EU/EEA countries, remains a persistent public health challenge in Romania, with outbreaks reported almost annually. In this study, we reviewed the main characteristics of the foodborne botulism outbreaks reported over the past 18 years to provide an overview of the disease and to propose evidence-based recommendations for strengthening national surveillance and control, as well as to raise awareness among clinicians on this pathology.

As anticipated, the majority of outbreaks were linked to the consumption of pork products, consistent with previous studies describing the association between botulism cases in Romania and traditionally prepared pork [6,16,22]. This meat is typically home cured through salting, smoking, aging, or preservation in lard, and consumed throughout the year [6,16].

According to the literature, the epidemiology of foodborne botulism varies across countries, reflecting how cultural food practices shape it [23]. However, our study highlights the fact that the situation varies within a single country, with only 25 counties out of the 42 reporting foodborne botulism outbreaks. Additionally, nearly half of all outbreaks and outbreak-related cases were reported in western Romania, particularly in Bihor, Satu Mare, Timiș and Arad. The persistent burden of foodborne botulism observed in western Romania has been previously reported and has been associated with traditional culinary practices and socio-economic factors [8,16,22,24]. Most outbreaks affected younger age categories, and our study identified a statistically significant age difference between sporadic and outbreak-related cases. This difference may be attributed to cultural and household practices common in Romania, where younger individuals are more frequently exposed to traditionally prepared foods consumed during family gatherings and shared meals [25]. In such settings, home-preserved products, often prepared using traditional methods, are shared with others, as this is a sign of hospitality and community bonding [26,27,28]. This hypothesis is further supported by the observed gender distribution of cases, which showed no statistically significant differences between sporadic and outbreak-related cases [29].

While higher percentages of sporadic cases were laboratory-investigated, a result which can be explained by the resource limitations and the operational consideration that exhaustive serological testing is often unnecessary during well-characterized outbreaks, type B toxin was detected for most cases, either sporadic or outbreak-related. Toxin E was identified in one case for which fish was identified as vehicle. According to a systematic review of foodborne botulism outbreaks reported between 1924 and 2014, the distribution of BoNT types varies across geographic regions, likely reflecting differences in food preferences and availability. In the United States and Asia, most outbreaks were attributed to toxin type A, whereas in Canada, type E dominated. In contrast, outbreaks reported in Europe and Africa were mostly associated with toxin types B or A. Food testing is crucial for outbreak control, confirming the presence of BoNT in suspected food, allowing safe removal of the vehicle and preventing the occurrence of additional cases. However, food testing results may be influenced by acidity, processing mechanisms, and storage conditions, which can reduce toxin detection. Differences in food type and preparation methods can also influence bacterial growth, and some tests may not detect all toxin types [30,31].

In terms of severity, a previously published study reported type B BoNT and prompt administration of antitoxin as associated with the lowest mean number of deaths [32]. Our findings are consistent with this study, considering the relatively low case-fatality ratio, particularly among outbreak-associated cases. Although treatment information was unavailable for all reported cases, the response measures implemented during the largest outbreak in 2024, consisting of a coordinated response for rapid procurement and administration of ABE antitoxin for all cases ensured by an interministerial organization, the Operational Center for Emergency Situations, as well as a legislative proposal to include a small quantity of ABE antitoxin in the anti-epidemic stockpile of every county indicate that, beyond the generally lower clinical severity linked to type B BoNT, timely administration of antitoxin played a crucial role in cases’ recovery. This applied to both sporadic and outbreak-related cases throughout the study period. These observations are in line with previous studies covering the surveillance periods 1990–2007 and 2010–2020, which reported the administration of ABE antitoxin to all foodborne botulism patients admitted to a single infectious disease hospital in western Romania, with complete recovery observed in 98% of cases [16,33].

In addition to the description of outbreaks by time, place, and person, our study highlights some challenges in the diagnosis, surveillance, and management of foodborne botulism in Romania. Notably, delayed recognition of early symptoms for the pediatric cases, as exemplified by the large outbreak in 2024, remains a major diagnostic challenge [8]. Delays in symptom recognition may result from the nonspecific nature of early clinical manifestations, as well as from limited clinical experience. Such delays can compromise timely treatment, hinder the identification and removal of the implicated vehicle and complicate traceback investigations. Consequently, the outbreak vehicle may remain undetected, as also observed in our study, thereby reducing the effectiveness of control measures [31]. Additionally, delayed identification of foodborne botulism cases, particularly sporadic cases, may result in negative laboratory results, which can lead to the misclassification of cases and an underestimation of the true disease burden [30,31]. Therefore, authorities should consider the influence on delayed diagnosis on laboratory findings when establishing case definitions for foodborne botulism. Our study results also emphasize the need to account for potential voluntary misreporting during outbreak investigations and to reassess epidemiological hypotheses when inconsistencies arise. Moreover, our findings highlight the importance of ensuring sufficient stocks of botulinum antitoxin, as outbreaks are reported almost annually in Romania and the number of cases may surpass expected levels. Finally, the study highlights the importance of standardized data collection and interoperable electronic databases to ensure long-term data traceability, as well as the importance of the training of healthcare professionals in ensuring adequate outbreak management.

Limitations: This was a retrospective study conducted over 18-year period, which limits the consistency and availability of surveillance data (missing information on the provenience of the vehicle, laboratory results for the vehicle, clinical presentation and management), which constrained the ability to perform more detailed analyses, including assessments of disease severity and treatment outcomes. Additionally, the burden of foodborne botulism may be underestimated considering the exclusion criteria.

Strengths: this study provides the first overview of foodborne botulism outbreaks in Romania, highlighting the burden of the disease as well as the challenges, gaps and achievements in its control.

## 5. Conclusions

In conclusion, our study highlighted that foodborne botulism remains a disease of major public health interest in Romania. To strengthen its control, we recommend enhancing clinician awareness, improving epidemiological investigation capacity, and establishing targeted communication campaigns. Key measures include implementing culturally tailored health education campaigns to promote safe food preparation and preservation, developing training activities on botulism management for healthcare professionals, establishing a centralized electronic case database, ensuring continuous antitoxin availability and fostering cross-disciplinarity and community partnerships. Enhancing the implementation of these measures in high-incidence areas could have the potential to reduce the burden of foodborne botulism in Romania.

## Figures and Tables

**Table 1:** Exposures and clinical characteristics of foodborne botulism cases by outbreak status, Romania, 2007–2024 (N = 337).

Variable	Overall, N = 337 ^1^	Sporadic, N = 190 ^1^	Outbreak-Related, N = 147 ^1^	*p*-Value ^2^
Age	41.0, 40.3 (27.0, 53.0)	45.0, 44.5 (33.0, 55.0)	31.0, 34.8 (23.0, 48.0)	<0.001
Sex				0.063
Male	119 (35%)	59 (31%)	60 (41%)	
Female	218 (65%)	131 (69%)	87 (59%)	
Number of days from onset to notification	6.0, 7.5 (4.0, 9.0)	7.0, 7.7 (4.0, 9.0)	6.0, 7.2 (4.0, 9.0)	0.078
Classification				0.13
Confirmed	303 (90%)	175 (92%)	128 (87%)	
Probable	34 (10%)	15 (8%)	19 (13%)	
Type of food				<0.001
Pork	249 (74%)	122 (64%)	127 (86%)	
Fish	58 (17%)	40 (21%)	18 (12%)	
Vegetables/fruits	30 (9%)	28 (15%)	2 (2%)	
Deaths (case fatality ratio)	15 (4%)	12 (6%)	3 (2%)	0.059

^1^ Median; Mean (IQR); n (%). ^2^ Wilcoxon rank sum test; Pearson’s Chi-squared test; Fisher’s exact test.

## Data Availability

The original contributions presented in this study are included in the article/Supplementary Material. Further inquiries can be directed to the corresponding author.

## Supplementary Materials

The following supporting information can be downloaded at https://www.mdpi.com/article/10.3390/microorganisms14040819/s1, Table S1. Main characteristics of foodborne botulism outbreaks, Romania, 2007–2024 (N = 55).

## References

[1] Chaidoutis E., Keramydas D., Papalexis P., Migdanis A., Migdanis I., Lazaris A.C., Kavantzas N. (2022). Foodborne botulism: A brief review of cases transmitted by cheese products (Review). Biomed. Rep..

[2] World Health Organization Botulism. https://www.who.int/news-room/fact-sheets/detail/botulism.

[3] Orphanet: Foodborne Botulism. https://www.orpha.net/en/disease/detail/228371.

[4] European Centre for Disease Prevention and Control (2024). Botulism.

[5] Meurice L., Filleul L., Fischer A., Burbaud A., Delvallez G., Diancourt L., Belichon S., Clouzeau B., Malvy D., Oliva-Labadie M. (2023). Foodborne botulism outbreak involving different nationalities during the Rugby World Cup: Critical role of credit card data and rapid international cooperation, France, September 2023. Euro Surveill..

[6] Păuna A.M., Crăciun M.D., Sîrbu A., Popescu R., Enciu B.G., Chivu C.D., Simoiu M., Pițigoi D. (2024). Botulism Cases in Romania-An Overview of 14-Year National Surveillance Data. Biomedicines.

[7] Anniballi F., Auricchio B., Fiore A., Lonati D., Locatelli C.A., Lista F., Fillo S., Mandarino G., De Medici D. (2017). Botulism in Italy, 1986 to 2015. Euro Surveill..

[8] Rao A.K., Lin N.H., Griese S.E., Chatham-Stephens K., Badell M.L., Sobel J. (2017). Clinical Criteria to Trigger Suspicion for Botulism: An Evidence-Based Tool to Facilitate Timely Recognition of Suspected Cases During Sporadic Events and Outbreaks. Clin. Infect. Dis..

[9] Government of Canada PHA Clostridium Botulinum: Infectious Substances Pathogen Safety Data Sheet. https://www.canada.ca/en/public-health/services/laboratory-biosafety-biosecurity/pathogen-safety-data-sheets-risk-assessment/clostridium-botulinum.html.

[10] Terranova W., Breman J.G., Locey R.P., Speck S. (1978). Botulism type B: Epidemiologic aspects of an extensive outbreak. Am. J. Epidemiol..

[11] Nishiura H. (2007). Incubation period as a clinical predictor of botulism: Analysis of previous izushi-borne outbreaks in Hokkaido, Japan, from 1951 to 1965. Epidemiol. Infect..

[12] Jackson K.A., Mahon B.E., Copeland J., Fagan R.P. (2015). Botulism mortality in the USA, 1975–2009. Botulinum J..

[13] Centurioni D.A., Egan C.T., Perry M.J. (2022). Current developments in diagnostic assays for laboratory confirmation and investigation of botulism. J. Clin. Microbiol..

[14] Hendrickx D., Varela Martínez C., Contzen M., Wagner-Wiening C., Janke K.H., Hernando Jiménez P., Massing S., Pichler J., Tichaczek-Dischinger P., Burckhardt F. (2023). First cross-border outbreak of foodborne botulism in the European Union associated with the consumption of commercial dried roach (Rutilus rutilus). Front. Public Health.

[15] Yang W., Jiang D., Li R., Sun L. (2023). Food-borne botulism from homemade sauce leading to cardiac arrest: A family case series with literature review. Toxicon.

[16] Neghina A.M., Marincu I., Moldovan R., Iacobiciu I., Neghina R. (2010). Foodborne botulism in southwest Romania during the post-communism period 1990–2007. Int. J. Infect. Dis..

[17] Government Resolution 589 from 13/06/2007 Regarding the Methodology for Collecting and Reporting Surveillance Data for Communicable Disease. https://monitoruloficial.ro/.

[18] Rafila A., Pitigoi D. (2012). Case Study–Romania. Biopreparedness and Public Health.

[19] Government Resolution 657 from 18/05/2022 Regarding the Methodology for Collecting and Reporting Surveillance Data for Communicable Disease in the National Registry of Communicable Diseases. https://monitoruloficial.ro/.

[20] Order of the Ministry of Health, Romania 1738 from 29/06/2022 Regarding the Methodology and Frequency of Reporting Data Based on Standardised National Unique form for Communicable Disease and Early Alert Sistems for Prevention and Control of Communicable Disease. https://monitoruloficial.ro/.

[21] National Institute of Public Health National Centre for Communicable Diseases Surveillance and Control. Methodological Frameworks. Botulism. https://insp.gov.ro/centrul-national-de-supraveghere-si-control-al-bolilor-transmisibile-cnscbt/metodologii/.

[22] Negrut N., Aleya L., Behl T., Diaconu C.C., Munteanu M.A., Babes E.E., Toma M.M., Bungau S. (2021). Epidemiology of botulism in the north-western Romania-a 7-year survey. Environ. Sci. Pollut. Res. Int..

[23] Heymann D. (2022). Control of Communicable Diseases Manual.

[24] Dragomir C.M., Ilie S.M., Poiana M.A., Misca C.D., Cocan I., Dumbrava D.G., Moldovan C., Popa V.M., Petcu C.D., Roman C. (2024). Traditional romanian culinary practices and their historical and cultural significance–A review. Sci. Papers Ser. E. Land Reclam. Earth Obs. Surv. Environ. Eng..

[25] Romania-Insider.com Survey Maps Patterns of Family Meals in Romania. https://www.romania-insider.com/family-meals-romania-2017.

[26] Tours in Romania A Culinary Journey: Dining with Local Families in Rural Romania or Bulgaria. https://www.tours-in-romania.com/blog/a-culinary-journey-dining-with-local-families-in-rural-romania-or-bulgaria.

[27] UntravelledPaths A Guide to the Romanian Christmas Food. https://untravelledpaths.com/blog/a-guide-to-the-romanian-christmas-food.

[28] Romania Guided Tours The Best Romanian Sausages and Meat-Based Products. https://romania-tours.travel/the-best-romanian-sausages-and-meat-based-products/.

[29] The Romanian Cookbook The Most Delicious Traditional Romanian Food. https://theromaniancookbook.com/traditional-romanian-food/.

[30] Lindström M., Korkeala H. (2006). Laboratory diagnostics of botulism. Clin. Microbiol. Rev..

[31] US Food and Drug Administration Fish and Fishery Products Hazards and Controls. https://www.fda.gov/food/seafood-guidance-documents-regulatory-information/fish-and-fishery-products-hazards-and-controls.

[32] Fleck-Derderian S., Shankar M., Rao A.K., Chatham-Stephens K., Adjei S., Sobel J., Meltzer M.I., Meaney-Delman D., Pillai S.K. (2017). The Epidemiology of Foodborne Botulism Outbreaks: A Systematic Review. Clin. Infect. Dis..

[33] Marincu I., Bratosin F., Vidican I., Cerbu B., Suciu O., Turaiche M., Tirnea L., Timircan M. (2021). Foodborne Botulism in Western Romania: Ten Years’ Experience at a Tertiary Infectious Disease Hospital. Healthcare.

