# Supplementary material for: Epidemiology of Foodborne Botulism Outbreaks in Romania, 2007–2024
Source: Microorganisms. 2026 Apr 2;14(4):819. doi: 10.3390/microorganisms14040819 (PMC13118966; doi:10.3390/microorganisms14040819)
Supplement: Supplementary file 1 [file microorganisms-14-00819-s001.zip › microorganisms-4204177-supplementary.pdf]

Supplementary material S1. Main characteristics of foodborne botulism outbreaks, Romania, 2007-2024 (N=55)

County	Year	Month	Suspected vehicle	Number of cases	Median age (years)	Median number of days onset-notification	Number of deaths
Arad	2007	June	pork	2	27	3	0
Arad	2010	December	pork	3	25	4	0
Arad	2011	August	pork	2	19	10	0
Argeș *(Outbreak 1)*	2009	February	pork	6	40	7	1
Bacău	2007	January	pork	2	36	8	0
Bihor	2007	July	pork	2	37	3	0
Bihor	2007	August	pork	2	22	13	0
Bihor	2010	January	pork	3	16	12	0
Bihor	2012	April	pork	3	44	3	0
Bihor	2012	May	pork	4	37	3	0
Bihor	2013	February	pork	4	32	5	0
Bihor	2013	October	pork	3	32	8	0
Bihor	2014	February	pork	2	54	4	0
Bihor	2015	June	pork	2	36	7	0
Bihor	2017	January	pork	2	23	16	0
Bihor	2018	February	pork	2	51	6	0
Bistrița-Năsăud	2012	August	pork	2	55	5	0
Bucharest	2008	October	fish	2	52	10	0
Bucharest	2019	May	pork	2	40	2	0
Caraș-Severin	2014	February	pork	2	54	18	0
Cluj	2008	September	pork	2	43	45	0
Cluj	2012	June	fish	2	70	11	0
Dâmbovița	2012	August	pork	2	26	3	0
Galați	2007	May	fish	3	9	7	0

Harghita	2011	January	pork	2	26	2	0
Harghita	2014	August	pork	2	34	7	0
Hunedoara	2008	June	pork	4	32	2	0
Ialomița	2022	November	vegetables/ fruits	2	38	6	0
Iași	2014	June	fish	2	17	9	0
Iași	2019	November	fish	2	68	2	1
Maramureș	2019	April	pork	3	23	9	0
Mureș	2018	April	pork	2	51	18	0
Neamț	2016	July	fish	2	43	4	0
Neamț	2024	September	pork	2	33	10	0
Prahova	2016	November	fish	3	40	0	0
Sălaj	2009	August	pork	4	38	4	0
Sălaj	2016	February	pork	2	35	16	0
Sălaj	2019	May	pork	3	42	1	0
Satu-Mare	2008	June	pork	4	31	5	0
Satu-Mare	2009	March	pork	2	28	2	0
Satu-Mare *(Outbreak 2)*	2009	May	pork	6	31	5	0
Satu-Mare	2013	July	pork	2	24	1	0
Satu-Mare	2014	April	pork	2	30	8	0
Satu-Mare	2015	March	pork	2	39	11	0
Satu-Mare	2024	September	pork	2	41	11	0
Sibiu	2022	January	pork	3	51	22	0
Teleorman	2009	September	pork	2	29	7	0
Timiș	2009	July	pork	2	29	10	0
Timiș	2010	May	pork	4	29	3	0
Timiș	2023	August	pork	4	38	9	0
Timiș *(Outbreak 3)*	2024	January	pork	8	6	5	0
Vâlcea	2007	April	pork	2	35	8	0

Vaslui	2008	June	pork	2	18	5	0
Vaslui	2013	May	fish	2	64	4	0
Vaslui	2018	October	pork	2	70	13	1
